# Supplementary material for: Spatial variation and determinants of mother and newborn skin-to-skin contact care practices in Ethiopia: A spatial and multilevel mixed-effect analysis
Source: PLoS One. 2024 Feb 23;19(2):e0297305. doi: 10.1371/journal.pone.0297305 (PMC10889646; doi:10.1371/journal.pone.0297305)
Supplement: Attachment — Submitted filename: Response to reviewers final.docx [file pone.0297305.s001.docx]

**Author response to Editor and reviewers**

**Dear Editor and Reviewers,**

Thank you very much for your email dated Nov 29, 12023 incorporating the insight of the editor and reviewer’s comments. We, the authors, would like to express our gratitude to you for the insightful and constructive review that has led to the great improvement of our paper entitled **“Spatial variation and determinants of mother and newborn skin-to-skin contact care practices in Ethiopia: A spatial and multilevel mixed-effect analysis.** “We declared that all the data underlying the results presented in the study are publicly available from the Measure DHS website: (<https://dhsprogram.com/data/dataset_admin/login_main.cfm>). We have carefully reviewed the comments given by the editor, and reviewers and revised the manuscript accordingly. Our responses are given in a point-by-point manner for the editor, and reviewers’ comments using the Author’s response to reviewer form. If you have any concerns to be addressed, we are happy to consider them.

Best regards!

**Version 1:** PONE-D-23-22844

Date: 12/14/2023

**Academic editor comments and respective author’s response**

**Editor comment 1:** Please ensure that your manuscript meets PLOS ONE's style requirements, including those for file naming. The PLOS ONE style templates can be found at [**https://journals.plos.org/plosone/s/file?id=wjVg/PLOSOne_formatting_sample_main_body.pdf**](https://journals.plos.org/plosone/s/file?id=wjVg/PLOSOne_formatting_sample_main_body.pdf) **and** [**https://journals.plos.org/plosone/s/file?id=ba62/PLOSOne_ formatting_ sample_ title_authors_affiliations.pdf**](https://journals.plos.org/plosone/s/file?id=ba62/PLOSOne_%20formatting_%20sample_%20title_authors_affiliations.pdf)

**AuthorsResponse:** Thanks very much for this comment. The whole part of the manuscript has been updated as per the PLOS ONE style templates.

**Editor comment 2.**  We note that you have indicated that data from this study are available upon request. PLOS only allows data to be available upon request if there are legal or ethical restrictions on sharing data publicly. For more information on unacceptable data access restrictions, please see <http://journals.plos.org/plosone/s/data-availability#loc-unacceptable-data-access-restrictions>.

In your revised cover letter, please address the following prompts:

a) If there are ethical or legal restrictions on sharing a de-identified data set, please explain them in detail (e.g., data contain potentially sensitive information, data are owned by a third-party organization, etc.) and who has imposed them (e.g., an ethics committee). Please also provide contact information for a data access committee, ethics committee, or other institutional body to which data requests may be sent.

b) If there are no restrictions, please upload the minimal anonymized data set necessary to replicate your study findings as either Supporting Information files or to a stable, public repository and provide us with the relevant URLs, DOIs, or accession numbers. For a list of acceptable repositories, please see <http://journals.plos.org/plosone/s/data-availability#loc-recommended-repositories>.

We will update your Data Availability statement on your behalf to reflect the information you provide.

**AuthorsResponse:** Thanks very much for this constructive comment. The comment has been accepted and revision has been made to the “Data availability statement” (please, look at the cover letter above).

**Editor comment 3** your ethics statement should only appear in the Methods section of your manuscript. If your ethics statement is written in any section besides the Methods, please move it to the Methods section and delete it from any other section. Please ensure that your ethics statement is included in your manuscript, as the ethics statement entered into the online submission form will not be published alongside your manuscript.

**AuthorsResponse:** Thanks a lot, dear editor, for this insightful comment. The ethics statements appear only in the Methods section of the manuscript (see page 10, lines 213-219).

**Editor comment 4:** Please include a separate caption for each figure in your manuscript.

**Authors Response:** Thanks very much dear editor for this helpful comment. A separate caption has been included for each figure.

**Editor comment 5:** We note that Figures 3,4and 5 in your submission contain [map/satellite] images which may be copyrighted. All PLOS content is published under the Creative Commons Attribution License (CC BY 4.0), which means that the manuscript, images, and Supporting Information files will be freely available online…

**Authors Response:** Thanks very much for this insightful comment. The maps we used in Figures 3,4, and 5 are freely available at: <https://africaopendata.org/dataset/ethiopia-shapefiles>.

**Reviewer #1 comments and an author response**

**Comments**

**Reviewer #1:** his study explored the spatial variation and determinants of mother and newborn skin-to-skin care practices in Ethiopia. The comments are as follows:

**Authors:** dear reviewer, we thank you heartily for your constructive and supportive comments.

**Comment #1:** There are some languages errors need to be revised in the manuscript.

**Authors Response:** Thanks very much for your comments, dear reviewer, as per your recommendation, corrective revisions were made in the revised manuscript.

**Comment#2:** The title of Tables 3 and 4 should be revised, do not start with Table-X shows.

**Authors Response:** Thanks very much for your comments, dear reviewer, as per your suggestion; correction was made in the revised manuscript.

**Reviewer #2:** This manuscript addresses a pertinent public health issue in the context of developing countries and especially in Ethiopia. The manuscript is very well written and analysed using sound statistical methods. Besides meeting other objectives, Interpolation was also done to depict whole country practices based on the sample selected, it was a good effort on part of the authors.

**Authors:** dear reviewer, we thank you heartily for your constructive and supportive comments.

**Comment 1:** Why the most recent data from EDHS 2019 not used

**Authors Response:** Thanks very much, for your concern, dear reviewer, and the reason why we didn’t use the EDHS 2019 is because it is a mini report and some of the data set was incomplete. For example, here in this study, **media exposure** was identified as a significant factor of Skin to skin contact care practice of mothers and newborns, but it was not reported in the EDHS 2019 data set.

**Comment 2:** Were all the assumptions from the mixed method analysis met with, explain in manuscript in both cases whether these conditions were met with or not

**Authors Response:** Thanks very much, dear reviewer, the comment was accepted; the correction was made in the revised manuscript. It is indicated on pages 7-8, from lines 150-156.

**Comment 3:** How was weighted average of the sampled mothers taken, mention in the methodology

**Authors Response:** Thanks very much, dear reviewer, we accept your suggestion, and correction was made in the revised manuscript, indicated on page 7 from lines 141-144.

Comment 4: Figure 3 needs to mention the footnotes the full form of LLR etc. Also explain how were log likelihood ratio range selected to be depicted in this figure? What is its significance?

**Authors Response:** Thanks very much for your concern, dear reviewer; we accepted your concern. Dear reviewer, if we are right, your concern is mainly about Fig 4, if so as per your concern, a correction was made in the revised manuscript, please, look at Fig 4.

**Comment 5:** Figure 4 should mention clearly in footnotes about hot and cold spots same as mentioned in lines 229 and 232 of this manuscript.

**Authors Response:** Thanks very much for your constructive comments, dear reviewer; we accepted your concern. A correction was made in the revised manuscript; please look at the manuscript on page 14, from lines 261-272.

**Comment 6:** Legend colors for cold and hot spots are not conspicuous; please make them look much better than they currently are.

**Authors Response:** Thanks very much for your insightful comments, dear reviewer; we accepted your comment. Please, look at the improved legend in Fig 3.

**Comment 7:** Language/typos/mistakes can be seen in isolated places like Akaike repeated in line 160, lines 129-30 repeated phrase. Although the expression of language is good overall yet it should be given a thorough keen look.

**Authors Response:** Thanks very much for your critical insight, dear reviewer, correction was made in the revised manuscript.
